# JunB Inhibits ER Stress and Apoptosis in Pancreatic Beta Cells

**DOI:** 10.1371/journal.pone.0003030

**Published:** 2008-08-21

**Authors:** Esteban N. Gurzov, Fernanda Ortis, Latifa Bakiri, Erwin F. Wagner, Decio L. Eizirik

**Affiliations:** 1 Laboratory of Experimental Medicine, Université Libre de Bruxelles (ULB), Brussels, Belgium; 2 Centro Nacional de Investigaciones Oncológicas (CNIO), Madrid, Spain; University of Bremen, Germany

## Abstract

Cytokines contribute to pancreatic β-cell apoptosis in type 1 diabetes (T1D) by modulation of β-cell gene expression networks. The transcription factor Activator Protein-1 (AP-1) is a key regulator of inflammation and apoptosis. We presently evaluated the function of the AP-1 subunit JunB in cytokine-mediated β-cell dysfunction and death. The cytokines IL-1β+IFN-γ induced an early and transitory upregulation of JunB by NF-κB activation. Knockdown of JunB by RNA interference increased cytokine-mediated expression of inducible nitric oxide synthase (iNOS) and endoplasmic reticulum (ER) stress markers, leading to increased apoptosis in an insulin-producing cell line (INS-1E) and in purified rat primary β-cells. JunB knockdown β-cells and *junB^−/−^* fibroblasts were also more sensitive to the chemical ER stressor cyclopiazonic acid (CPA). Conversely, adenoviral-mediated overexpression of JunB diminished iNOS and ER markers expression and protected β-cells from cytokine-induced cell death. These findings demonstrate a novel and unexpected role for JunB as a regulator of defense mechanisms against cytokine- and ER stress-mediated apoptosis.

## Introduction

Diabetes mellitus is characterized by high levels of blood glucose resulting from defects in insulin production, insulin action or both. The two main forms of diabetes are type 1 (T1D) and type 2 diabetes (T2D). Both types are characterized by progressive pancreatic β-cell loss, which is more marked in T1D [1]. β-cell death in T1D is mediated by a protracted autoimmune attack where apoptosis is triggered by cytokines and by other mechanisms such as Fas-FasL or granzyme B [2], [3]. Previous microarray studies from our group indicate that β-cells up- and down-regulate complex gene networks in response to cytokines [4], [5]. This altered gene expression profile is regulated by key transcription factors such as NF-κB and STAT-1 [1], [5] and is responsible for activation of pro-apoptotic execution pathways like endoplasmic reticulum (ER) stress, resulting in β-cell dysfunction and death [6]. Of note, β-cells seem to have insufficient defense responses against some of these pro-inflammatory and pro-apoptosis pathways [7]–[9]. As a whole, these observations indicate that signaling events occurring inside the β-cells are decisive for their survival or death in diabetes.

The Activator Protein-1 (AP-1) transcription factor is a key player in response to extracellular signals, including cytokines [10]. AP-1 is composed of a variety of dimers of JUN, FOS, ATF and MAF families. These components share biochemical properties but differ in their biological functions. AP-1 can induce cell survival or death under stress conditions, and it is the balance between the regulation of pro- and anti-apoptotic target genes which determines the cell outcome. This balance varies from cell to cell, and depends on both the activated AP-1 member(s) and on the parallel regulation of other transcription factors [11]. Previous studies from our group indicate that the AP-1 subcomponent JunB is preferentially activated in human and rodent β-cells after cytokine exposure [4], [12], [13], but the role of this protein remains unclear. Here we investigated the effects of reducing or increasing JunB levels in insulin-producing INS-1E cells and in FACS-purified rat primary β-cells. We demonstrate that JunB counteracts cytokine-induced ER stress and apoptosis and might therefore be part of defense mechanisms triggered by β-cells during exposure to pro-inflammatory cytokines. Modulating JunB could represent a novel strategy to ameliorate β-cell resistance to immune damage.

## Methods

### Cell culture, treatments and nitric oxide (NO) measurement

Pancreatic islets were isolated from adult Wistar rats (Charles River Laboratories Belgium, Brussels, Belgium), housed and used according to the guidelines of the Belgian Regulations for Animal Care; the Ethical Committee for Animal Experiments of the ULB approved the experimental protocols. Islets were isolated by collagenase digestion, β-cells were purified by autofluorescence-activated cell sorting (FACS, FACStar, Becton-Dickinson and Co.; Sunnyvale, CA, USA) and pre-cultured for 48 h in HAM's F-10 medium [14]. Insulin-producing INS-1E cells [15] were cultured in RPMI-1640 medium (Invitrogen, Paisley, UK) supplemented with 5% FCS. 3T3 *junB* ^−/−^ and 3T3 wild-type mouse fibroblasts [16] were cultured in Dulbecco's modified Eagle's medium (DMEM) supplemented with 10% FCS. Recombinant rat or mouse IFN-γ (100–500 U/ml, R&D Systems, Abingdon, UK) and human recombinant IL-1β (10–50 U/ml, gift from Dr. C.W. Reinolds, National Cancer Institute, Bethesda, MD, USA), and mouse TNF-α (1000 U/ml, Innogenetics, Gent, Belgium) were used. The NO blocker L-NMA (NG-methyl-L-arginine, Sigma, Steinheim, Germany) was used at 2.5 M. Culture medium was collected for nitrite determination (nitrite is a stable product of NO oxidation) by the Griess method [4]. SP600125 and cyclopiazonic acid (CPA) (Sigma) were dissolved in DMSO and used at a concentration of 10 µM and 25 µM respectively. Cytokines, L-NMA, and CPA concentrations were selected based on our previous time course and dose-response studies [2], [4], [12].

### Infection with recombinant adenoviruses

After 48 h pre-culture cells were infected either with AdLUC (control luciferase expressing virus), AdIκB^(SA)2^ (encoding a previously described NF-κB super-repressor protein [17]) or AdJunB (expressing the rat JunB protein, purchased from RIKEN Bio Resource Center-Japan). Primary rat β-cells and INS-1E cells were infected for 2 h at 37°C with a MOI of 10 for AdIκB^(SA)2^ or AdLUC in INS-1E cells, and with a MOI of 20 and 40 for AdJunB and AdLUC in the INS-1E and primary β-cells, respectively.

### Western blotting

Equal amounts of proteins were resolved by 10% SDS-PAGE. Immunoblot analysis was performed with antibodies against JunB, IkBα, inducible nitric oxide synthase (iNOS) and Chop (Santa Cruz Biotechnology, CA, USA), p-Jun N-terminal Kinase (JNK) and β-actin (Cell Signaling, Danvers, MA, USA) and α-tubulin (Sigma). The proteins were detected using the corresponding horseradish peroxidase-conjugated secondary antibody (Santa Cruz Biotechnology), and chemiluminescence Supersignal (Pierce, Rockford, IL, USA) quantified using Aida1D analysis software (Fujifilm). Values of the protein of interest were corrected for β-actin or α-tubulin. All Western blots shown in the figures are representative of at least 2–3 experiments.

### Cell death assay

The percentage of cell death was determined by two observers, one of whom was unaware of sample identity, in ≥600 cells in each experimental condition by inverted fluorescence microscopy after addition of the DNA dyes Hoechst 33342 (20 µg/ml) and propidium iodide (10 µg/ml) (HO/PI) [4]. This method is quantitative and has been validated in pancreatic β-cells by systematic comparisons with electron microscopy [18], determination of DNA strand breaks [19] and caspase-3 activation [5].

### siRNA transfection

Cells were transfected overnight with 30 nM of siRNAs against JunB or Chop (sequences are provided in Table S1) or medium GC content inactive control siRNA (Invitrogen) using 1 µl of DharmaFECT™ lipid reagent (Dharmacon, Chicago, IL, USA) to 150 nM of siRNA in Opti-mem^R^ (Invitrogen). After overnight incubation, transfection medium was replaced by regular culture medium for cell recovery.

### Real Time RT-PCR

mRNA expression was determined by real time RT-PCR using SYBR Green fluorescence on a LightCycler instrument (Roche, Manheim, Germany). Primer sequences for mouse and rat iNOS, ATF4, BiP, XBP-1s and Chop are provided in Table S2. Expression of the gene of interest was divided by the house keeping gene glyceraldehyde-3-phosphate dehydrogenase (GAPDH) and expressed as fold induction compared to control. GAPDH expression is not modified under the present experimental conditions (data not shown).

### Chromatin immunoprecipitation (ChIP) assay

ChIP assays were performed as described [20]. Extracts were precleared by 1 h incubation with protein A/Herring sperm DNA, and IP was performed by incubating overnight at 4°C with the JunB antibody (sc-73, Santa Cruz Biotechnology) or using preimmune serum as negative control. Primer sequences for the AP-1 sites within rat iNOS and Chop promoter are provided in Table S3.

### Statistical analysis

Data are shown as means±SEM, and comparisons between groups were made by paired *t* test or by ANOVA followed by *t* test with Bonferroni correction for multiple comparisons.

## Results and Discussion

**Figure 1:**
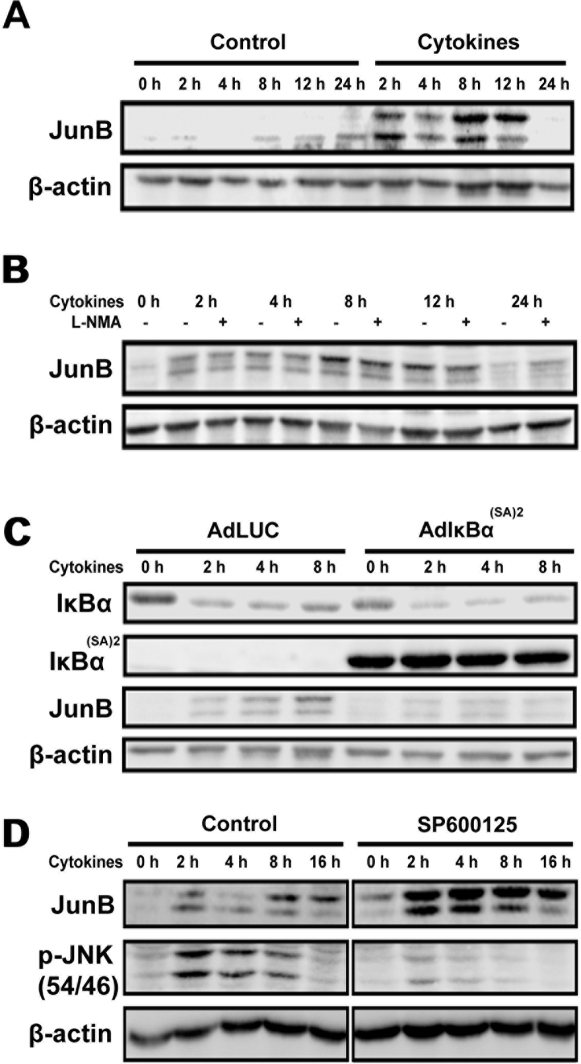
Cytokine-induced JunB upregulation in INS-1E cells is mediated via NF-κB activation. (A) INS-1E cells were treated with IL-1β+IFN-γ and JunB protein expression was determined by Western blot at different time points. The two observed bands correspond to JunB and its phosphorylated form. (B) JunB expression after cytokines and/or L-NMA exposure. (C) INS-1E cells were infected with a recombinant adenovirus expressing either luciferase (AdLUC) or the NF-κB nondegradable inhibitor IkB^(SA)2^ (AdIκB^(SA)2^) and then exposed to IL-1β+IFN-γ. Expression of IκBα, IκBα^(SA)2^ and JunB were determined by Western blot. (D) INS-1E cells were treated with cytokines (IL-1β+IFN-γ) and/or the JNK inhibitor SP600125 at different time points as indicated, JunB and p-JNK expression were measured by Western blot.

JunB protein expression and its phosphorylation were increased in insulin producing INS-1E cells as early as 2 hours after cytokine addition. This lasted for up to 12 h, returning to basal levels after 24 h (Fig. 1A). NO generated by the inducible form of iNOS is an important mediator of cytokine-driven changes in β-cell gene expression [4]. JunB induction was not affected by the NO blocker L-NMA, indicating a direct cytokine effect independent of NO generation (Fig. 1B). NF-κB regulates JunB expression in fibroblasts and endothelial cells [21], [22]. Infection of INS-1E cells with the adenovirus AdIκB^(SA)2^ expressing an IκB “super-repressor” prevented cytokine-induced NF-κB nuclear translocation (Fig. S1) and abolished JunB induction (Fig. 1C), indicating that cytokine-mediated JunB activation in β-cells is regulated by NF-κB. It has been shown that JNK regulates the turnover of JunB through its accelerated degradation induced by JNK-mediated phosphorylation of the E3 ligase Itch [23]. To examine whether JNK affects JunB stability, we exposed INS-1E cells to cytokines and/or the JNK inhibitor SP600125. We observed that reducing cytokine-mediated JNK activation leads to increased JunB protein expression (Fig. 1D), in agreement with data in other cell types [23], [24].

**Figure 2:**
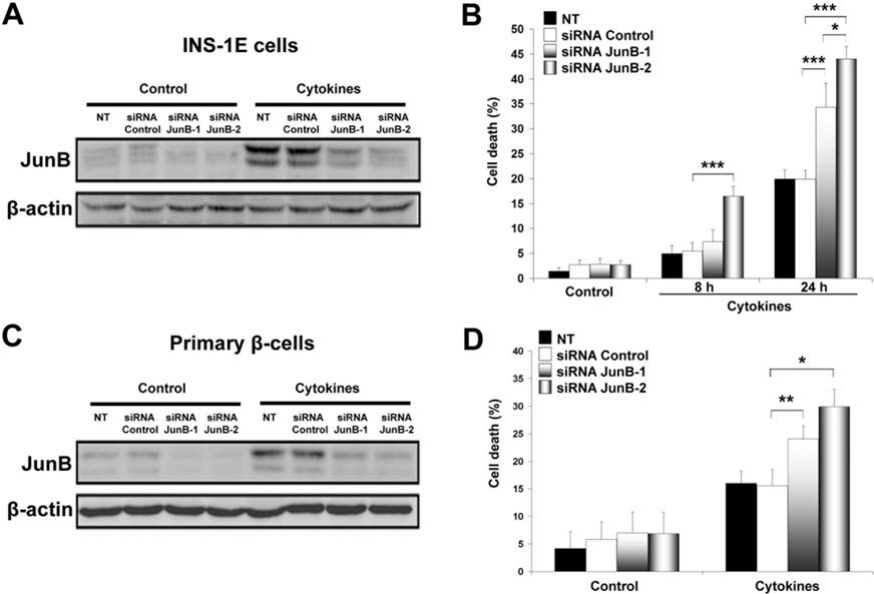
JunB knockdown augments cytokine-induced β-cell death. (A) INS-1E cells were transfected with inactive (Control) or two different active JunB siRNAs; Western blot for JunB was performed 8 h after cytokine exposure. Non-transfected cells (NT) were used as additional control. (B) Cytokines were added for 8 or 24 h to INS-1E cells and cell death was measured by HO/PI. (C) Purified rat β-cells were transfected with control or with JunB siRNAs, and Western blot for JunB was performed 8 h after cytokine addition. (D) JunB knockdown increases primary β-cell death 48 h after cytokine treatment. Results are the means±SEM of 3-5 independent experiments. *P<0.05, **P<0.01, ***P<0.001.

To study the role of JunB in cytokine-mediated effects, we used RNAi technology to selectively knockdown JunB. Two different JunB siRNAs inhibited JunB protein expression by >65% as compared with an inactive control siRNA in INS-1E cells (Fig. 2A, quantification in Fig. S2). JunB knockdown did not affect viability under basal conditions, but it increased cytokine-induced apoptosis by 2-fold at 24 h (Fig. 2B). The siRNA JunB-2, which induced a more marked knockdown effect, increased cytokine-mediated β-cell death to a larger extent. In line with these observations, primary β-cells were also sensitized to cytokine-induced apoptosis after JunB knockdown (Fig. 2C,D). Previous studies have shown that a *junB* transgene prevents liver apoptosis in *c-jun^−/−^* embryos [25], while JunB inactivation induces cell death in c-Jun knockout fibroblasts [24]. To the best of our knowledge, there are no previous reports demonstrating a role of this protein in cytokine-mediated apoptosis.

**Figure 3:**
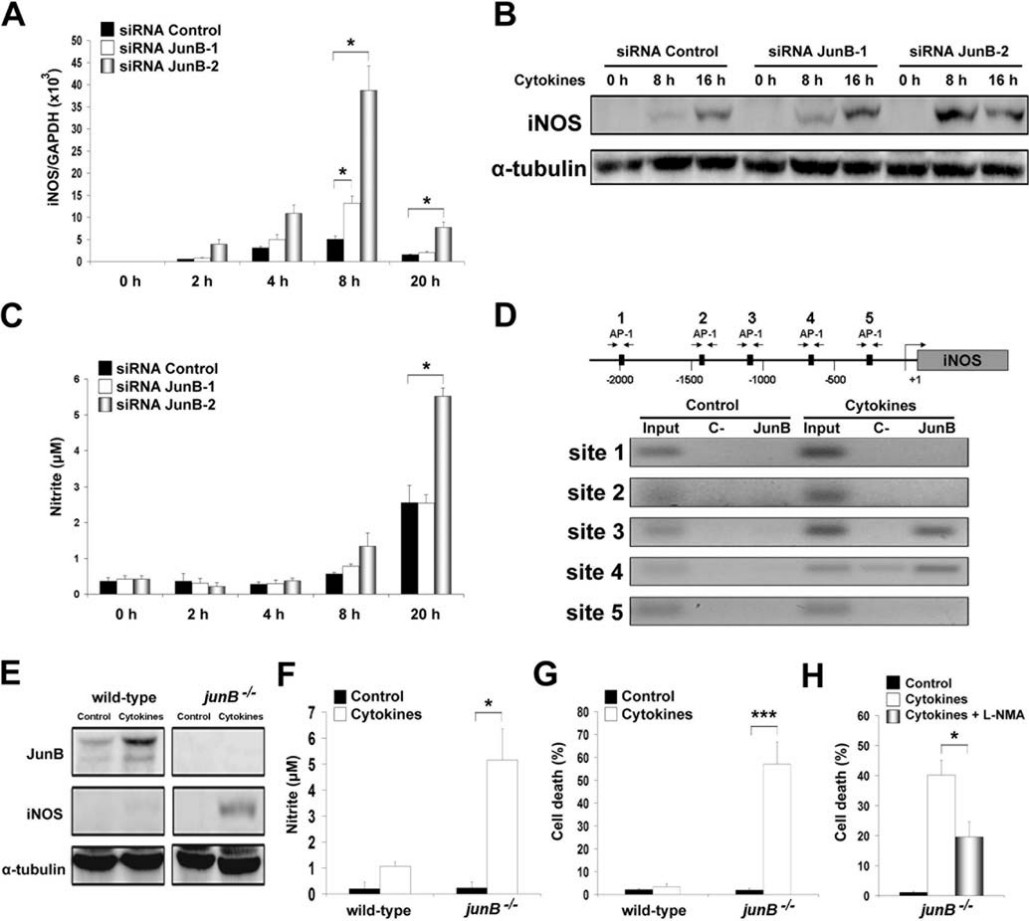
JunB inactivation upregulates cytokine-induced iNOS expression. (A) INS-1E cells were transfected with control or JunB siRNAs and treated with cytokines. iNOS expression was examined at different time points by real time RT-PCR. (B) Western blot demonstrating increased expression of iNOS protein in the cytokine-treated JunB knockdown cells. (C) Nitrite accumulation in the culture medium. (D) Schematic representation of the rat iNOS promoter with putative AP-1 binding sites and the localization of the primers used. ChIP reveals binding of JunB to the iNOS promoter 4 h after cytokine treatment in two of the five putative AP-1 binding sites as assessed by PCR. (E) Wild-type or *junB^−/−^* fibroblasts were treated with cytokines (IL-1β+IFN-γ+TNF-α) for 24 h, JunB and iNOS expression were measured by Western blot. (F) Increased nitrite production by JunB knockout fibroblasts. (G) Cell death in fibroblasts as measured by HO/PI. (H) Cytokine-induced cell death in *junB^−/−^* fibroblasts was partially prevented by L-NMA. Results are the means±SEM of 3 independent experiments. *P<0.05.

Cytokine-induced iNOS expression in β-cells increases NO production, which has been shown to contribute to β-cell death [2]. JunB knockdown potentiated iNOS mRNA and protein induction after cytokine treatment (Fig. 3A,B). Consistently, higher levels of the NO product nitrite were detected in the culture medium of cytokine-treated JunB knockdown cells (Fig. 3C). iNOS production is primarily controlled at the transcriptional level [26] and the *iNOS* promoter contains several binding sites for AP-1 [27], [28]. ChIP analysis using a JunB antibody demonstrated JunB binding to two of the five different putative AP-1 binding sites in the *iNOS* promoter (Fig. 3D). These data indicate that JunB might act as a direct transcriptional repressor of *iNOS* expression. To determine whether the effects of JunB are specific for pancreatic β-cells, we exposed wild-type or JunB knockout fibroblasts to cytokines. The treatment markedly increased iNOS expression and NO production leading to cell death in JunB knockout fibroblasts, but not in wild-type cells (Fig. 3E–G). Furthermore, L-NMA attenuated cytokine-induced apoptosis in *junB^−/−^* fibroblasts (Fig. 3H), suggesting that NO contributes for cell death in this model and that JunB prevents cytokine-mediated iNOS expression and NO toxicity in different cell types.

**Figure 4:**
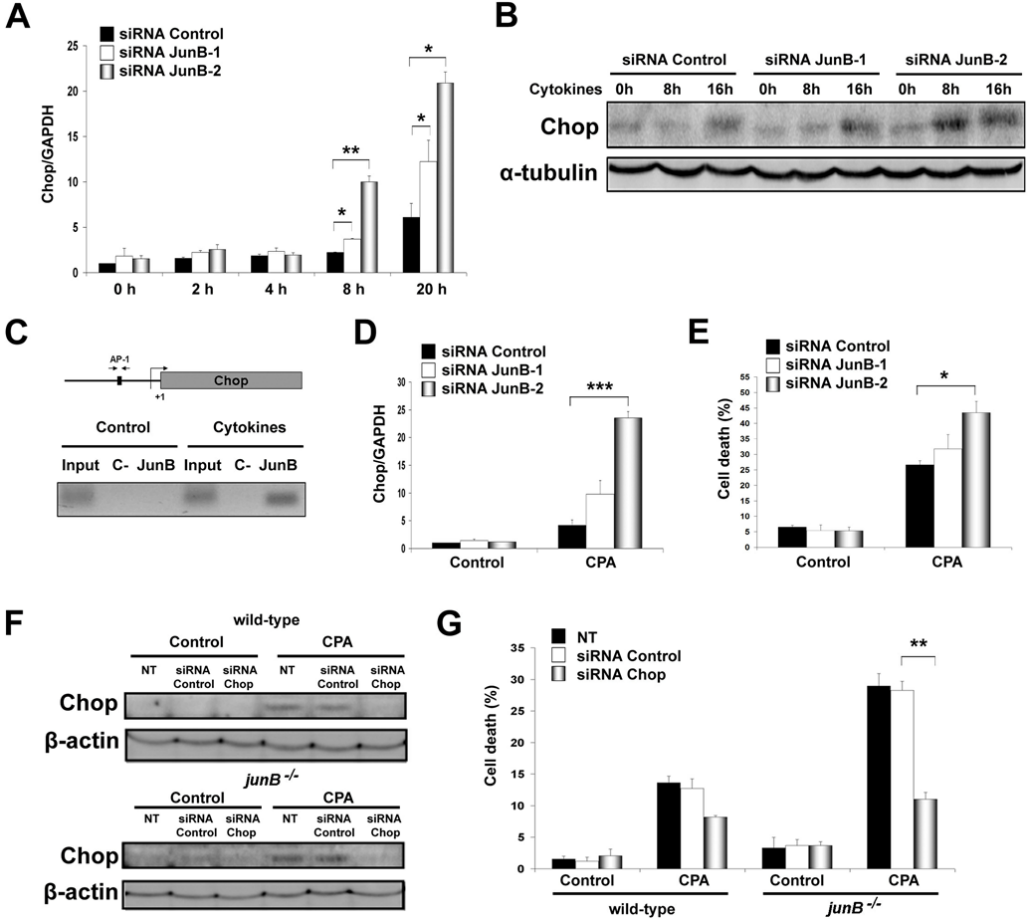
Chop expression is upregulated in JunB knockdown and cytokine-treated cells. (A) RT-PCR of INS-1E cells transfected with control or JunB siRNAs and treated with cytokines. (B) Increased Chop protein expression after JunB knockdown and cytokine treatment was confirmed by Western blot. (C) Ethidium bromide-stained agarose gel of PCR products obtained with primers flanking the AP-1 binding site in the *Chop* promoter. After cytokine treatment, IP was done for JunB or control (−). (D) Chop mRNA expression is increased in JunB knockdown β-cells 15 h after the CPA (25 µM) addition. (E) Cell death in siRNA transfected INS-1E cells treated with CPA. (F) Fibroblasts were transfected with control or Chop siRNAs and Western blot for Chop was performed 24 h after CPA addition. Non-transfected cells (NT) were used as an additional control. (G) siRNA-mediated Chop knockdown decreased fibroblast cell death 24 h after CPA addition. Results are the means±SEM of 3 independent experiments. *P<0.05, **P<0.01, ***P<0.001.

**Figure 5:**
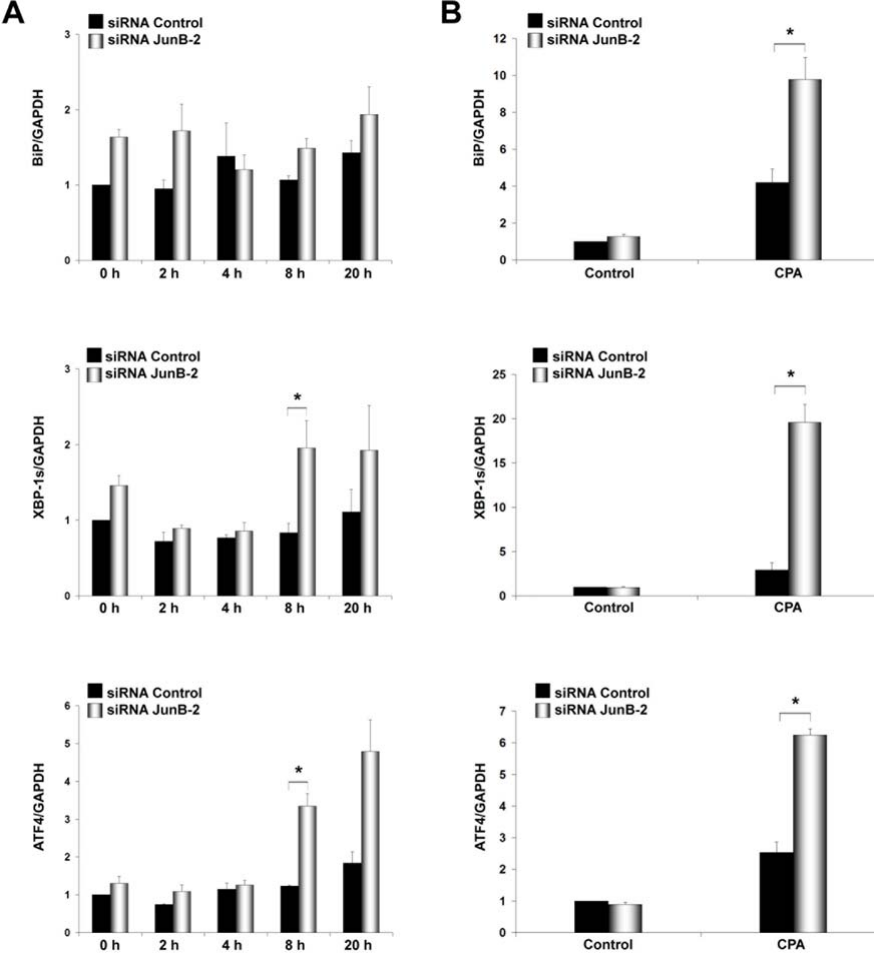
Real time RT-PCR for ER stress markers in INS-1E cells after cytokines addition at the indicated time points (A) or 15 h CPA treatment (B). Results are the means±SEM of 3 independent experiments. *P<0.05.

**Figure 6:**
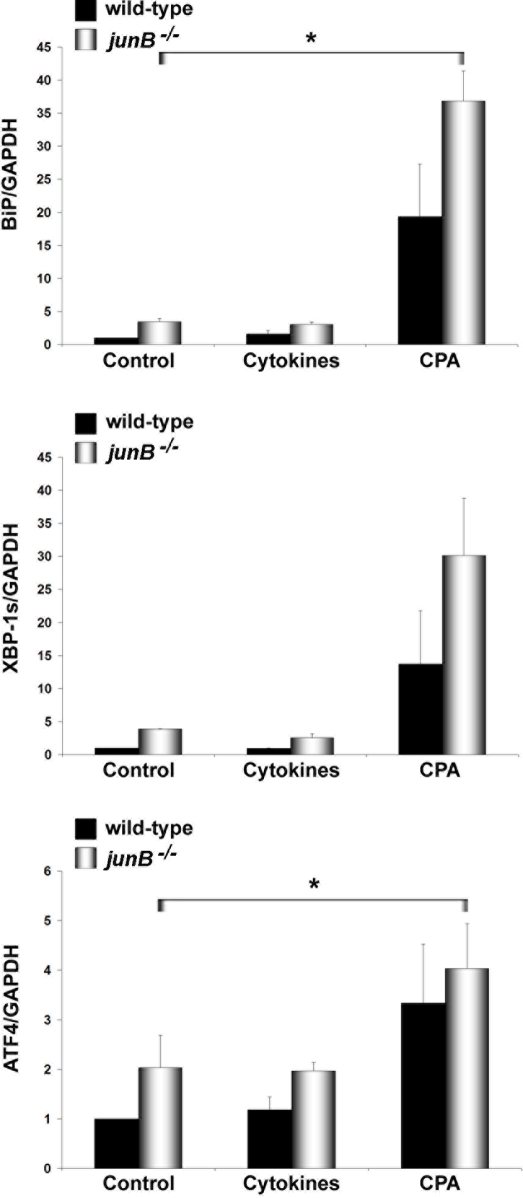
Real time RT-PCR for ER stress markers in fibroblasts 24 h after cytokine or CPA treatment. Results are the means±SEM of 3 independent experiments. *P<0.05.

IL-1β+IFN-γ decrease the expression of the sarco(endo)plasmic reticulum calcium ATPase (SERCA2) in β-cells via NO synthesis, thereby depleting ER Ca^2+^ stores and causing ER stress [29]. The transcription factor Chop is a key regulator of ER stress–mediated apoptosis [6], [30], [31]. Cytokines induce Chop expression in β-cells, and this was shown to be NO- and AP-1-dependent [12]. JunB knockdown induced an early upregulation of Chop mRNA and protein after cytokine addition (Fig. 4A,B). ChIP assay using primers designed to amplify the unique AP-1 site in the regulatory sequence of the *chop* gene [12] indicated strong JunB binding after cytokine treatment (Fig. 4C). This confirms physical interaction of JunB with the *chop* promoter, and suggests that JunB negatively regulates Chop expression, in agreement with our previous observation that mutation of the AP-1 site in the *chop* promoter increases basal activity, presumably by dissociating an inhibitory binding complex [12]. We next exposed INS-1E cells transfected with JunB siRNAs or fibroblasts lacking JunB to the chemical ER stressor CPA, a SERCA2 blocker that induces Chop and ER stress-mediated cell death independently of NO [12]. Addition of CPA increased Chop expression and apoptosis in JunB knockdown β-cells (Fig. 4D,E). Interestingly, *junB^−/−^* fibroblasts were also more sensitive to CPA-induced apoptosis and increased Chop mRNA and protein expression compared to wild-type cells (Fig. S3A–C). Chop expression was regulated at the promoter level as indicated by experiments using a *Chop* promoter luciferase reporter construct (Fig. S3D). These data indicate that JunB negatively modulates ER stress-induced Chop expression independently of NO induction, presumably via direct transcriptional repression. Chop knockdown by siRNA prevented CPA-induced apoptosis in *junB^−/−^* fibroblasts (Fig. 4F,G), indicating that Chop is a crucial mediator of ER stress-induced apoptosis. Importantly, JunB inactivation also increased expression of the ER stress markers BiP, XBP-1s and ATF4 after cytokine (INS-1E cells) or CPA treatment (INS-1E cells and fibroblasts) (Figs. 5 and 6) suggesting that all major pathways of the ER stress response are negatively regulated by JunB.

**Figure 7:**
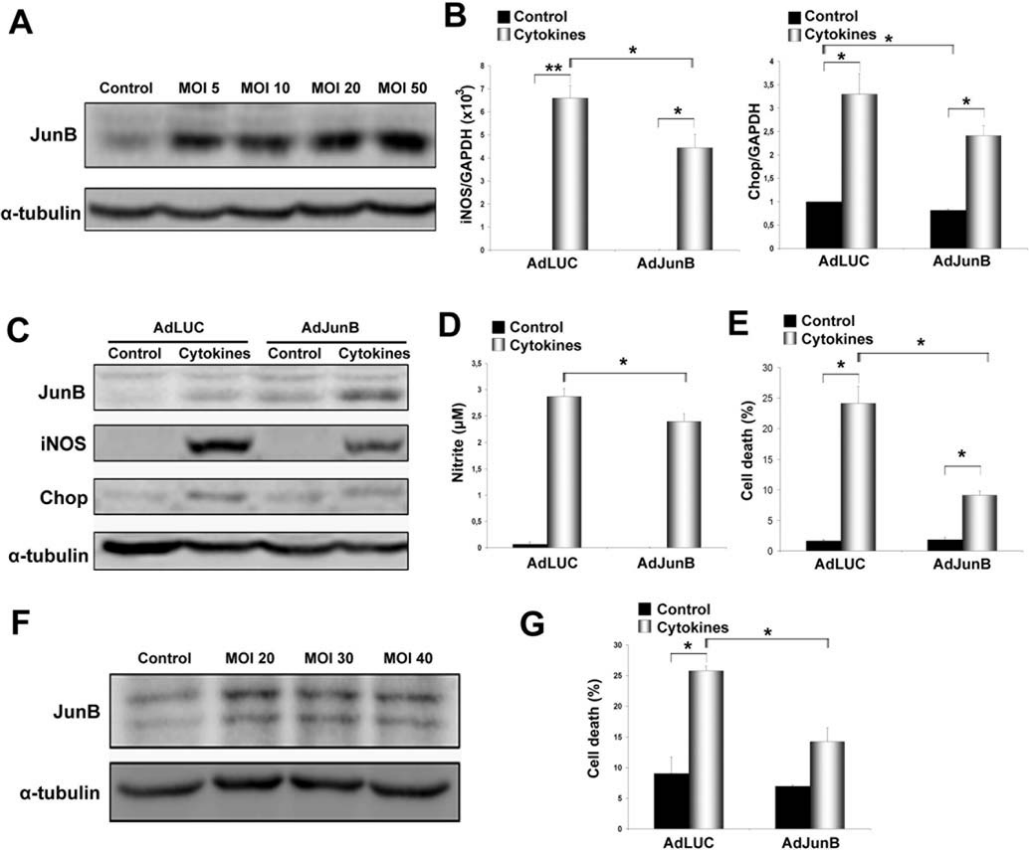
Adenovirus-mediated overexpression of JunB protects INS-1E and primary β-cells from apoptosis after cytokine treatment. (A) Western blot showing JunB upregulation in INS-1E cells 24 h following infection with AdJunB at different MOIs. (B) iNOS and Chop mRNA expression in adenovirus-infected and cytokine-treated INS-1E cells was analyzed by real time PCR. (C) Western blot showing upregulation of JunB and downregulation of Chop and iNOS 24 h after infection of INS-1E cells with AdJunB compared with AdLUC (control) virus. (D) Nitrite accumulation in the medium of cells used in A. (E) Adenovirus-infected cells were exposed to cytokines for 24 h and cell death was measured with HO/PI. (F) Western blot showing JunB upregulation 48 h after infection of primary β-cells with AdJunB at different MOIs. (G) Percentage of cell death (HO/PI) after cytokine treatment in primary β-cells infected with AdJunB or control AdLUC. Results are the means±SEM of 3 independent experiments. *P<0.05, **P<0.01.

Since JunB knockdown or knockout aggravated ER stress and apoptosis, we next examined whether overexpression of JunB protects β-cells from cytokine-induced cell death. JunB was overexpressed using an adenoviral vector containing the rat JunB cDNA [32] under the control of the CAG promoter [33]. JunB protein expression was efficiently increased in INS-1E cells at the different multiplicity of infections (MOIs) tested (Fig. 7A); we selected MOI 20 for subsequent experiments. JunB overexpression reduced cytokine-mediated iNOS and Chop induction (Fig. 7B,C), and diminished nitrite production (Fig. 7D). In addition, INS-1E cells infected with AdJunB and exposed to cytokines or CPA displayed significantly decreased BiP but not XBP-1s or ATF4 expression (Fig. S4). Most importantly, prolonged JunB overexpression protected both INS-1E and primary β-cells against cytokine-induced apoptosis (Fig. 7E–G).

**Figure 8:**
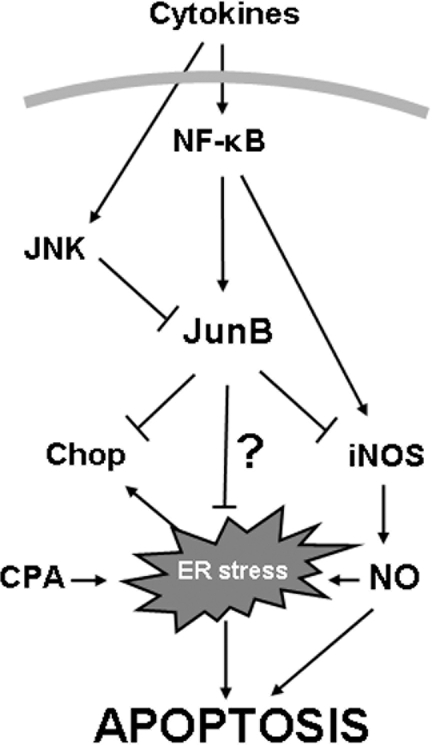
Proposed model for the role of JunB in cytokine- and CPA-mediated ER stress response. Cytokines induce a transitory JunB expression via NF-κB activation. JunB has an inhibitory role on iNOS and Chop expression and on other pro-ER stress and pro-apoptotic pathways that remain to be determined, favoring β-cell survival. Following prolonged exposure to cytokines, however, JunB turnover is induced by JNK and the pro-apoptotic effects of cytokines prevail. Prolonged overexpression of JunB using adenoviral vectors decreases cytokine-induced ER stress and β-cell apoptosis.

Taken together, our data show a cytokine-mediated early upregulation of JunB in β-cells. JunB induction is controlled by NF-κB, while its late return to base line is probably secondary to cytokine-triggered JNK activation and subsequent JunB degradation. It is thus conceivable that the previously reported pro-apoptotic role of JNK in cytokine-treated β-cells [34], [35] is at least partially mediated by JNK-induced JunB degradation (present data). It is noteworthy that while adenoviral-sustained overexpression of JunB protects β-cells against cytokine-mediated apoptosis (Fig. 7), endogenous cytokine-induced JunB expression fails to prevent cell death. This is probably related to the transitory nature of JunB expression, which peaks around 8 h and returns to baseline by 24 h after cytokine addition (Fig. 1), the time point when a significant increase in apoptosis is observed (Fig. 2B). Our results are in line with previous reports indicating that β-cell sensitivity to cytokine-induced cell death is aggravated by its feeble and/or transitory induction of protective genes such as XIAP [36], Bcl-6 [7] or suppressor of cytokine signaling (SOCS)-3 [37]. We show that JunB is a key regulator of the cytokine-induced ER stress response in β-cells. There are conflicting data, however, on whether cytokine-induced ER stress is relevant for β-cell apoptosis. Thus, while some studies indicate that upregulation of the chaperone BiP [38] or blocking Chop [6], [31] prevents cytokine-induced β-cell death, other studies failed to observe a protective effect of chemical chaperones and siRNA targeting Chop [39] or described a dissociation between IL-1 induced ER stress and caspase-3 activity [40]. As described in Fig. 8, the protective mechanism of JunB in cytokine-induced β-cell apoptosis is mediated, at least in part, via inhibition of iNOS and ER stress markers expression. However, it is likely that JunB also regulates other pro- or anti-apoptotic pathways that remain to be determined.

In conclusion, we demonstrate that JunB plays an unexpected protective role against cytokine-induced apoptosis in pancreatic β-cells. Knockdown of this AP-1 component aggravates cytokine-mediated cell death, whereas JunB overexpression protects β-cells from apoptosis. These findings suggest that increasing JunB expression could improve β-cell survival in early diabetes.

## Supporting Information

Figure S1 Cells were plated in poly-lysine coated cover slips and, after 30 min treatment with cytokines, fixed with 4% paraformaldehyde and permeabilized with 70% acetone+30% methanol. Cells were then incubated for 1 h with anti-p65 antibody (sc-372, Santa Cruz Biotechnology) at 1:500 dilution. The secondary antibody FITC conjugated anti-rabbit IgG (Jackson ImmunoResearch; diluted 1:200) was used for visualization by inverted fluorescence microscopy (Zeiss Axiovert 200, Oberkochen-Germany). NF-κB activation was evaluated by presence of its p65 subunit in the nucleus. We have previously shown that p65 is the main constituent of NF-κB in cytokine-treated β-cells (Ortis F, Cardozo AK, Crispim D, Storling J, Mandrup-Poulsen T, et al. (2006) Cytokine-Induced Proapoptotic Gene Expression in Insulin-Producing Cells Is Related to Rapid, Sustained, and Nonoscillatory Nuclear Factor-κB Activation. Mol Endocrinol 20: 1867–1879).
(0.34 MB JPG)

Figure S2 Densitometry quantification of three experiments performed as shown in Figure 2A. *P<0.05, **P<0.01.
(0.37 MB TIF)

Figure S3 (A) Apoptosis is strongly induced by CPA in JunB KO fibroblasts. Wild-type or junB-/- fibroblasts were treated for 24 h with CPA (25 µM) and cell death was measured by HO/PI. Increased Chop mRNA (B) and protein (C) expression in JunB KO fibroblasts after CPA-induced ER stress was measured by respectively RT-PCR and Western blot. (D) The hamster Chop promoter (−782 to 21 bp) tethered to a luciferase reporter gene was used [12]. Fibroblasts were transfected using lipofectAMINE 2000 (Invitrogen) with 250 ng of the luciferase reporter construct and 50 ng of the pRL-CMV plasmid (used as internal control for transfection efficiency). Twenty-four hours after transfection cells were exposed for 24 h to CPA. Luciferase activities of cell lysates were determined and expressed as Firefly/Renilla (relative luciferase activity). *P<0.05, **P<0.01, ***P<0.001.
(0.48 MB TIF)

Figure S4 Real time RT-PCR for ER stress markers in INS-1E after AdLUC or AdJunB infection and subsequent 24 h cytokine or CPA treatment. Results are the means±SEM of 3–6 independent experiments. *P<0.05, **P<0.01, ***P<0.001.
(0.38 MB JPG)

Table S1 siRNA sequences for protein knockdown.
(0.03 MB DOC)

Table S2 Primer sequences for real time RT-PCR.
(0.03 MB DOC)

Table S3 Primer sequences for analysis of JunB binding to the rat iNOS and Chop promoters in the ChIP studies performed.
(0.03 MB DOC)
