# Supplementary figures and images for: JunB Inhibits ER Stress and Apoptosis in Pancreatic Beta Cells
Source: PLoS One. 2008 Aug 21;3(8):e3030. doi: 10.1371/journal.pone.0003030 (PMC2516602; doi:10.1371/journal.pone.0003030)

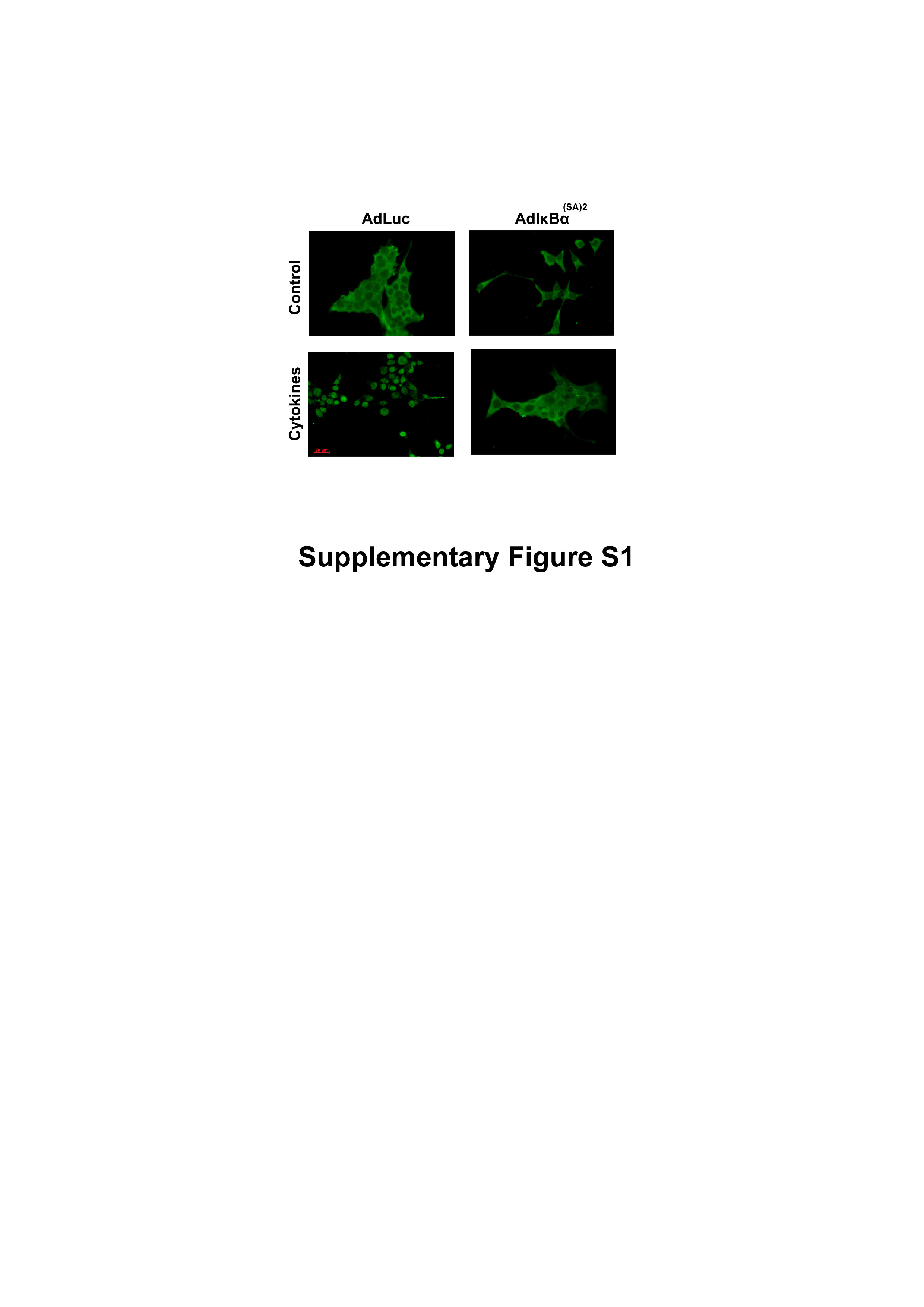

Supplement: Figure S1 — Cells were plated in poly-lysine coated cover slips and, after 30 min treatment with cytokines, fixed with 4% paraformaldehyde and permeabilized with 70% acetone+30% methanol. Cells were then incubated for 1 h with anti-p65 antibody (sc-372, Santa Cruz Biotechnology) at 1:500 dilution. The secondary antibody FITC conjugated anti-rabbit IgG (Jackson ImmunoResearch; diluted 1:200) was used for visualization by inverted fluorescence microscopy (Zeiss Axiovert 200, Oberkochen-Germany). NF-κB activation was evaluated by presence of its p65 subunit in the nucleus. We have previously shown that p65 is the main constituent of NF-κB in cytokine-treated β-cells (Ortis F, Cardozo AK, Crispim D, Storling J, Mandrup-Poulsen T, et al. (2006) Cytokine-Induced Proapoptotic Gene Expression in Insulin-Producing Cells Is Related to Rapid, Sustained, and Nonoscillatory Nuclear Factor-κB Activation. Mol Endocrinol 20: 1867–1879). (0.34 MB JPG) [file pone.0003030.s001.jpg]

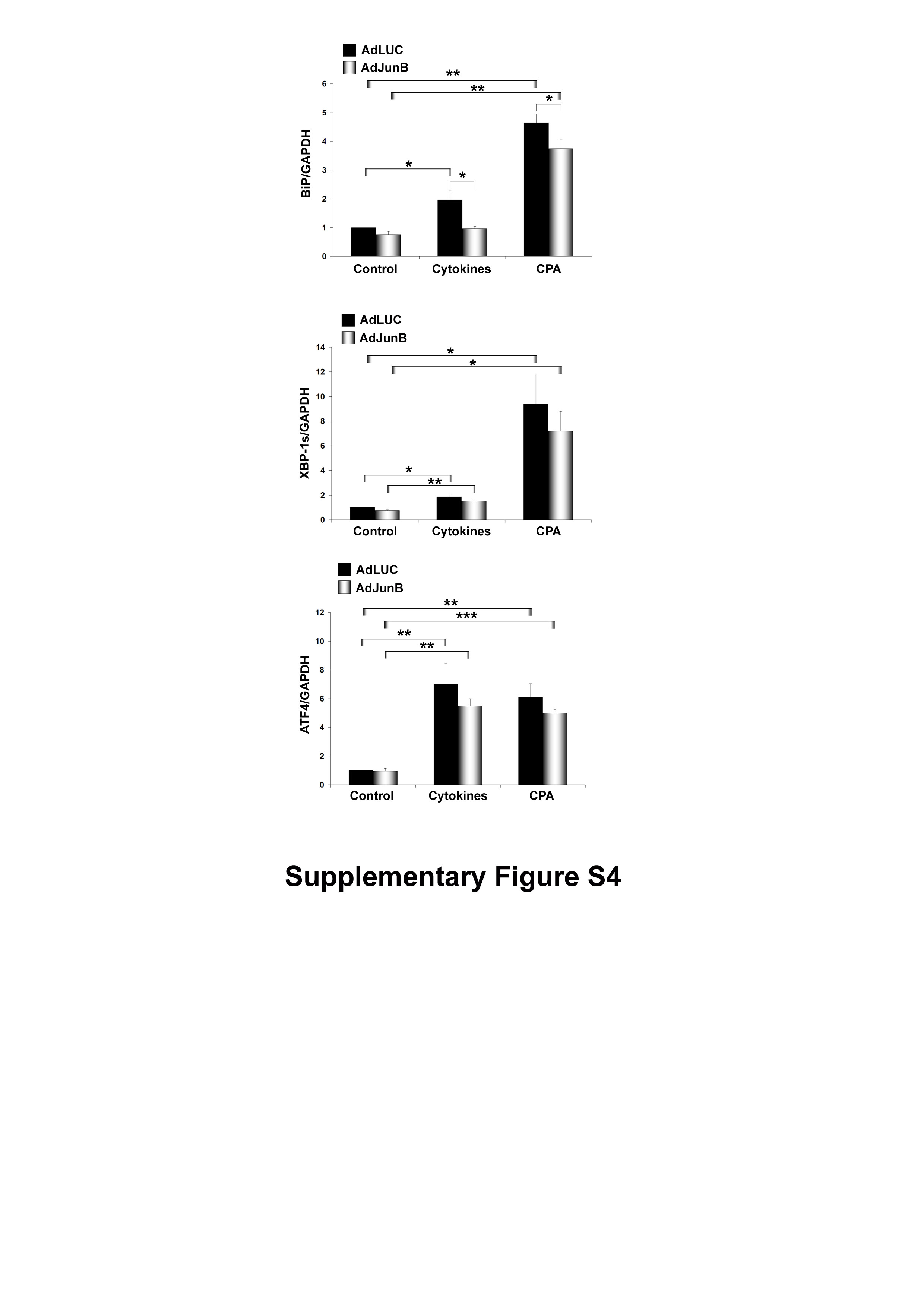

Supplement: Figure S4 — Real time RT-PCR for ER stress markers in INS-1E after AdLUC or AdJunB infection and subsequent 24 h cytokine or CPA treatment. Results are the means±SEM of 3–6 independent experiments. *P<0.05, **P<0.01, ***P<0.001. (0.38 MB JPG) [file pone.0003030.s004.jpg]
