# Supplementary material for: JunB Inhibits ER Stress and Apoptosis in Pancreatic Beta Cells
Source: PLoS One. 2008 Aug 21;3(8):e3030. doi: 10.1371/journal.pone.0003030 (PMC2516602; doi:10.1371/journal.pone.0003030)
Supplement: Table S1 — siRNA sequences for protein knockdown. (0.03 MB DOC) [file pone.0003030.s005.doc]

**Supplementary Table S1** siRNA sequences for protein knockdown.

| **Name** | **Sequence** |
| --- | --- |
| siRNA JunB-1 | 5’-AUGAGUCGUCGUGAUAGAAAGGCUG-3’ |
| siRNA JunB-2 | 5’-AGGAGCGCAUCAAAGUGGAGCGAAA-3’ |
| siRNA Chop | 5’-GGAAGAACUAGGAAACGGAtt-3’ |
