# Supplementary material for: JunB Inhibits ER Stress and Apoptosis in Pancreatic Beta Cells
Source: PLoS One. 2008 Aug 21;3(8):e3030. doi: 10.1371/journal.pone.0003030 (PMC2516602; doi:10.1371/journal.pone.0003030)
Supplement: Table S2 — Primer sequences for real time RT-PCR. (0.03 MB DOC) [file pone.0003030.s006.doc]

**Supplementary Table S2** Primer sequences for real time RT-PCR.

| **Name** | **Forward** | **Reverse** |
| --- | --- | --- |
| Bip  (mouse and rat) | 5’-CCACCGGATGCAGACATTG-3’ | 5’-AGGGCCTCCACTTCCATAGA-3’ |
| Chop  (mouse and rat) | 5’-GTCTCTGCCTTTCGCCTTTG-3’ | 5’-CTACCCTCAGTCCCCTCCTC-3’ |
| ATF4  (mouse and rat) | 5’-TCCTGAACAGCGAAGTGTTG-3’ | 5’-CGCACTGACCACTCTGTTTC-3’ |
| XBP-1s  (mouse) | 5’-GAGTCCGCAGCAGGTG-3’ | 5’-GCGTCAGAATCCATGGGA-3’ |
| XBP-1s  (rat) | 5’-GAGTCCGCAGCAGGTG-3’ | 5’-GTGTCAGAGTCCATGGGA-3’ |
| iNOS  (rat) | 5’-GGGAGCCAGAGCAGTACAAG-3’ | 5’-GGCTGGACTTCTCACTCTGC-3’ |
