# Supplementary material for: JunB Inhibits ER Stress and Apoptosis in Pancreatic Beta Cells
Source: PLoS One. 2008 Aug 21;3(8):e3030. doi: 10.1371/journal.pone.0003030 (PMC2516602; doi:10.1371/journal.pone.0003030)
Supplement: Table S3 — Primer sequences for analysis of JunB binding to the rat iNOS and Chop promoters in the ChIP studies performed. (0.03 MB DOC) [file pone.0003030.s007.doc]

**Supplementary Table S3** Primer sequences for analysis of JunB binding to the rat *iNOS* and *Chop* promoters in the ChIP studies performed.

| **Name** | **Forward** | **Reverse** |
| --- | --- | --- |
| AP-1 site 1 *iNOS* | 5’-AGTATCTGTTTCACAGAGTCC-3’ | 5’-CACTAGGTTTAGAAGTTTGCC-3’ |
| AP-1 site 2 *iNOS* | 5’-ATTATAAGTGTGTGTCACCAC-3’ | 5’-TGTACCACTGAGCATGCATGC-3’ |
| AP-1 site 3 *iNOS* | 5’-GCAAGGCAAGCACTTTGACG-3’ | 5’-AAAGTGTGTGGTGTGGCCTC-3’ |
| AP-1 site 4 *iNOS* | 5’-GAAGTAAAGGCGTGTGCCAC-3’ | 5’-AGTAAATTAGTTTGGGCAGC-3’ |
| AP-1 site 5 *iNOS* | 5’-GTGGTGCAGCTAAGAAAAGC-3’ | 5’- TGTATCTTCGGTGAGGCCAC-3’ |
| AP-1 site *Chop* | 5’-CGCGCATGACTCACTCAC-3’ | 5’- GCCACTCAGGAGTCCCGT -3’ |
